# Frailty and risk of delirium among UK Biobank participants

**DOI:** 10.1002/alz.093360

**Published:** 2025-01-09

**Authors:** Ruixue Cai, Chenlu Gao, Xi Zheng, Kun Hu, Lei Gao, Peng Li

**Affiliations:** ^1^ Massachusetts General Hospital, Boston, MA USA; ^2^ Brigham and Women’s Hospital, Boston, MA USA

## Abstract

**Background:**

Delirium is a common neurological complication in older adults after a major illness or surgery and is characterized by acute dysfunction in cognition, attention, and awareness. Once diagnosed, it is associated with an increased risk for dementia. Frailty is an age‐related syndrome and is associated with poorer postoperative outcomes, and is an emerging risk factor for delirium. The current study aimed to examine the association between frailty and delirium risk in middle‐ to older‐aged adults.

**Method:**

We examined frailty and incident delirium in 389,040 UK Biobank participants (38‐73 years old; 178,377 females) were hospitalized at least once during follow‐up. Frailty at baseline was assessed using two validated measures: (1) frailty phenotype (FP) that is identified when three or more of the following five criteria were fulfilled: self‐reported weight loss, exhaustion, low physical activity, slow walking pace, and low grip strength; (2) frailty index (FI: range 0‐1) that is calculated from 49 items covering health, presence of diseases and disabilities, and mental well‐being (FI score ≥0.24 indicating frailty). A new onset of delirium was obtained from hospitalization records using ICD‐10 F05 coding. Cox proportional hazards models assessed the association between frailty and delirium.

**Result:**

At baseline, 4.3% (15,311/359,979) and 5.2% (20,061/389,040) were identified as frail cases using FP and FI, respectively. The participants had been followed for a median of 12 years, with 6,710 newly developed delirium cases. Compared with non‐frail participants based on FP, participants with frailty were at a higher risk for delirium after adjusting for age, sex, ethnicity, and education (Hazard ratio [HR] = 3.22, 95% CI = 2.97‐3.49, *p*<0.0001). Similar results were observed based on FI, i.e., frail participants had an adjusted HR of 2.79 (95% CI = 2.59‐3.00, *p*<0.0001) for delirium compared with non‐frail participants. These observations were consistent after further adjusting for social economic status, body mass index, sleep duration, smoking, and alcohol consumption (HR = 2.48, 95% CI = 2.28‐2.70 for FP; HR = 2.17, 95% CI = 2.01‐2.35 for FI, both *p* values <0.0001).

**Conclusion:**

Frailty predicted delirium in middle‐ to older‐aged adults. Assessment and management of frailty may help with risk stratifications for delirium.

